# Exploring the Relationship Between Public Social Media Accounts, Adolescent Mental Health, and Parental Guidance in England: Large Cross-Sectional School Survey Study

**DOI:** 10.2196/57154

**Published:** 2024-12-17

**Authors:** Wakithi Siza Mabaso, Sascha Hein, Gabriela Pavarini, The OxWell Study Team, Mina Fazel

**Affiliations:** 1 Department of Psychiatry University of Oxford Oxford United Kingdom; 2 Department of Clinical Child and Adolescent Psychology University of Giessen Giessen Germany; 3 Department of Education & Psychology Freie Universität Berlin Berlin Germany; 4 Child Study Center Yale University New Haven, CT United States; 5 Ethox Centre Oxford Population Health University of Oxford Oxford United Kingdom; 6 Department of Social Policy and Intervention University of Oxford Oxford United Kingdom; 7 see Acknowledgments

**Keywords:** social media, adolescent health, privacy, parental guidance, mood disorders, adolescent, anxiety, depression, cross-sectional, mental health, public, account, school-going, school, England, survey, logistic regression, observational

## Abstract

**Background:**

Although associations between social media use and adolescent mental health have been described, more information is needed on the potential components characterizing this complex exposure, in particular, those related to maintaining a public social media account.

**Objective:**

This study aims to investigate the association between having a public social media account and anxiety and depression in school-going adolescents.

**Methods:**

Overall, 80 secondary schools and further education colleges in England were sampled using a cross-sectional web-based survey as part of the 2023 OxWell Student Survey. Social media exposure was categorized among the adolescents as having a public social media account versus not having a public social media account. The risk of clinical anxiety and depression was determined using the Revised Child Anxiety and Depression Scale-11. Adolescents self-reported the content and platforms accessed in the previous 24 hours. Associations between having a public social media account and symptoms of anxiety and depression were assessed using logistic regression controlling for age, sex, the experience of being bullied, parental guidance of online behavior (describing perceived parental approaches to adolescents’ online activity), the proportion of close friendships engaged with online, poverty status, and placement in statutory care. Age, sex, and parental guidance of online behavior were assessed for primary association effect modification.

**Results:**

Data collected from 16,655 adolescents (aged 11-18 y) were analyzed. Of these 16,655 adolescents, 6734 (40.43%) had a public social media account, while 9921 (59.57%) either had a private social media account or no social media account. Moreover, 32.6% (5429/16,655) of the adolescents screened positive for symptoms of anxiety and depression. Those with a public social media account had higher odds of anxiety and depression (odds ratio [OR] 1.41, 95% CI 1.32-1.50) than those without a public social media account in an unadjusted and fully adjusted model (OR 1.39, 95% CI 1.29-1.49). Adolescents reporting active parental guidance had lower odds of anxiety and depression (OR 0.85, 95% CI 0.75-0.93) than those reporting no parental guidance, and these parental approaches to online behaviors significantly modified the association between having a public social media account and symptoms of anxiety and depression (*P*=.004; χ^2^_2_=11.1).

**Conclusions:**

Our OxWell study findings suggest a potential mental health risk for adolescents with a public social media account. We show evidence indicating some protection from anxiety and depression among adolescents who do not have a public social media account and those reporting some form of parental guidance of their online behavior. This was pronounced in adolescents reporting active parental guidance compared to stricter regulatory approaches or no guidance at all. The specific roles that social media account choices and parental guidance of online behavior may play in supporting the mental health of adolescents are highlighted for further investigation.

**International Registered Report Identifier (IRRID):**

RR2-10.1136/bmjopen-2021-052717

## Introduction

### Background

Associations between social media use and adolescent mental health have been described; however, new characterizations of social media use are required to advance a nuanced understanding of this complex exposure, prompted by concerns over adolescents’ susceptibilities on social media platforms (SMPs). Existing findings draw on more than a decade of broad research into time spent on social media and online engagement patterns, including both active and passive social media use [1-3]. The rapidly changing nature of social media related behaviors exacerbates the difficulty of inquiry in this field and necessitates focused social media research questions to try to extend and explain the findings from existing studies to explore this complexity in greater depth.

One such area broadly concerns online privacy behaviors on social media through a simple characterization: having a public social media account versus not having a public social media account (ie, having a private social media account). Social media privacy behavior refers to actions or settings that allow users to control whom they communicate and share information with directly [4]. Adolescents can express such behaviors or phenotypes in the following ways: having a public social media account, not having a public social media account (ie, having a private social media account), having both a public social media account and a private one, and having no social media account at all [5]. In practice, social media privacy settings extend further to a set of complex parameters, including who can contact a user, customizable information disclosure, privacy concerns, the transparency of users’ online behavior to other users in their network, and the accessibility of their information to organizations and platforms through data-sharing agreements. Exercising their social media privacy by this behavioral definition facilitates young people’s expectations of SMPs and confers subjective control over the information they share and access. Understanding the differences between having a public social media account and not having one offers insights into the potential risks on adolescent mental health outcomes conferred by social media exposure, where private social media accounts require a follow request and permissions to access (and interact with) the user’s profile and content, while publicly available social media accounts do not [5].

### Prior Findings

#### Overview

Broadly, cross-sectional studies on social media and mental health outcomes have predominantly examined the duration of time spent online, finding significant associations between spending ≥2 hours on SMPs a day and depressive symptoms in adolescent girls [6-8], with discrete windows of sensitivity emerging early in boys (aged 14 y and 19 y) and in girls (aged 13-15 y and 19 y) [9]. Longitudinal studies on social media use and adolescent mental health have shown conflicting results. The UK Household Longitudinal Study of 3228 adolescents looked at the association between the duration of time spent on SMPs at ages 12 to 13 years and predicted mental health problems 2 years later at ages 14 to 15 years using the Strengths and Difficulties Questionnaire, a brief screening measure of behavioral and emotional problems, and found no association between time spent on SMPs and mental health problems [10]. A Swedish study of 3502 students found that adolescents who spent more time on social media reported higher levels of mental health problems measured using the Strengths and Difficulties Questionnaire [11]. In contrast to these findings, a US study of 388 adolescents using live mobile device use measurements and the Positive and Negative Affect Schedule for Children showed that adolescents’ daily duration of time spent on entertainment content was associated with less same-day worry and that sending more text or online messages per day was associated with lower depression scores [12].

The Stanford Social Media Lab conducted a systematic review and meta-analysis of 226 peer-reviewed papers involving 257,728 participants; the researchers demonstrated an overall null effect, indicating that social media use was not associated with well-being in bidirectional analysis [2]. However, analysis looking exclusively at negative indicators of well-being showed a small significant association between greater social media use and greater anxiety and depression symptoms. Analysis looking only at positive indicators of well-being also showed a small significant association, this time between greater social media use and greater social well-being symptoms. The presence of small significant associations in both negative and positive well-being indicators explained their aggregated null effect [2], and cross-lagged effects analysis showed a solitary, significant, unidirectional association between positive psychological well-being and lower social media use [2]. These findings hint at underlying drivers of heterogeneity, prompting the investigation of adolescents’ social media use and their potential effect modifiers, including parental approaches to adolescents’ online behaviors measured by adolescents' self-reported perceptions.

#### Parental Guidance of Online Behavior

Parental approaches to the guidance of young people’s online activities are suspected to predict positive mental health because parents who demonstrated awareness of their children’s activities significantly halved their children’s odds of reporting negative moods and feelings [13]. The type of web-related parental guidance reported by adolescents significantly influences their online privacy behaviors [14,15], and established social media privacy behaviors among young people are shown to be even more influential than their personality traits in predicting future privacy-related choices [15].

#### Social Media Privacy

Regarding social media privacy, Barsova et al [8], demonstrated that participants who attempted to exercise greater control over the social media material they were exposed to by using tools such as “unfollow” and managing notification settings to control the content in their feeds showed significantly lower levels of anxiety, depression, and stress. These actions resembled adolescents’ greater concern for their personal privacy behaviors online [7,13,15]. These associations illuminate gaps for further work strengthening the characterization and description of associations between social media privacy and mental health outcomes. The findings by Gruzd and Hernández-García [5] demonstrated distinct patterns between online privacy concerns and the protective posting behaviors of a subset of individuals who reported having both a public social media account and a private one but showed no intraindividual differences in protective posting behaviors across a given individual’s public and private accounts. Few studies have characterized interindividual differences between people with a public social media account and those without one. This highlights the importance of research investigating social media account types and their influence on mental health as a primary level of distinction between appreciably nuanced privacy behaviors and in keeping with a complex understanding of social media use behaviors [8].

### Relevance

With significant heterogeneity in the literature, it is suggested that newer characterizations of social media use, including social media privacy, SMP use, and content consumption, are useful toward a greater understanding of the specific behaviors from which effects on mental health may be expected [8]. This is important because of the rapid integration of social media with normative development in adolescents, who, in some aspects of their development, need to be considered a vulnerable group [16]. This developmental group vulnerability calls for the consideration of an ethical framework of accountability as it relates to the SMPs’ instrumentalization of content and users’ data against arising conflicts with adolescents’ social media use patterns and observed associations with their mental health outcomes per the goals of the Lancet Commission on Adolescent Health and Wellbeing [16].

Using data from the 2023 OxWell Student Survey, this study aims to explore the relationship between social media account privacy (ie, whether an adolescent has a public social media account versus whether they do not have a public social media account) as a behavioral proxy and their mental health outcomes across this behavior in a sample of English school-going adolescents. We ask whether there is a difference in the symptoms of anxiety and depression between adolescents who have a publicly available social media account and those who do not have a publicly available social media account (ie, have a private account). Next, we describe the distribution of SMPs and content accessed by adolescents, and finally, we discuss some of the ethical considerations of SMPs and their accountability surrounding adolescents as a vulnerable group.

### Hypotheses

The following hypotheses are examined:

- Small but significant associations are described between positive psychological well-being and decreased social media use, a potential proxy for agency over one’s social media exposure, which may extend to privacy behaviors [2]. Therefore, we hypothesize (H1) that the odds of anxiety and depression in adolescents with a publicly available social media account are higher than the odds of anxiety and depression in adolescents without a publicly available social media account.
- Adolescent privacy behaviors are shown to be influenced by an open discussion with adults, a component of parental guidance of online behavior [14], and prior work looking at age and sex differences in mental illness describes higher rates of anxiety and depression in young adolescent girls compared to boys [17]. Therefore, we hypothesize (H2) that the strength of the association between social media account type and anxiety and depression is modified by age, sex, and parental guidance of online behavior [14,17].

## Methods

### Study Characteristics

The OxWell Student Survey is a repeated cross-sectional survey of schools and further education colleges (FECs) in England, asking students various questions relating to their mental health and well-being, life experiences, and behaviors [18]. In 2023, it had 2 age-appropriate versions (divided into English school years 5 to 6 [primary] and 7 to 13 [secondary], collectively covering ages 9 to 18 years). This observational study used only secondary school and FEC data because the questions of interest were only asked to the older students. Participants came from 80 secondary schools and FECs, primarily in 4 regions in England: Oxfordshire, Berkshire, Liverpool, and Milton Keynes. The OxWell Student Survey 2023 (administered in February-March 2023) collected 42,215 valid consenting responses from 43,735 survey log-ins. Of the 42,215 valid consenting responses, 32,965 (78.09%) were from students in school years 7 to 13 (ages 11-18 y) in 80 secondary schools and FECs. The proportion of students who, upon logging in to the survey, declined to consent to participate was 3.48% (1520/43,735). Information on school-recorded opt-outs effected by nonconsenting parents was not shared with the study team in keeping with the study’s data protection protocols and ethics guidelines [18].

### Participant Sampling

**Figure 1:**
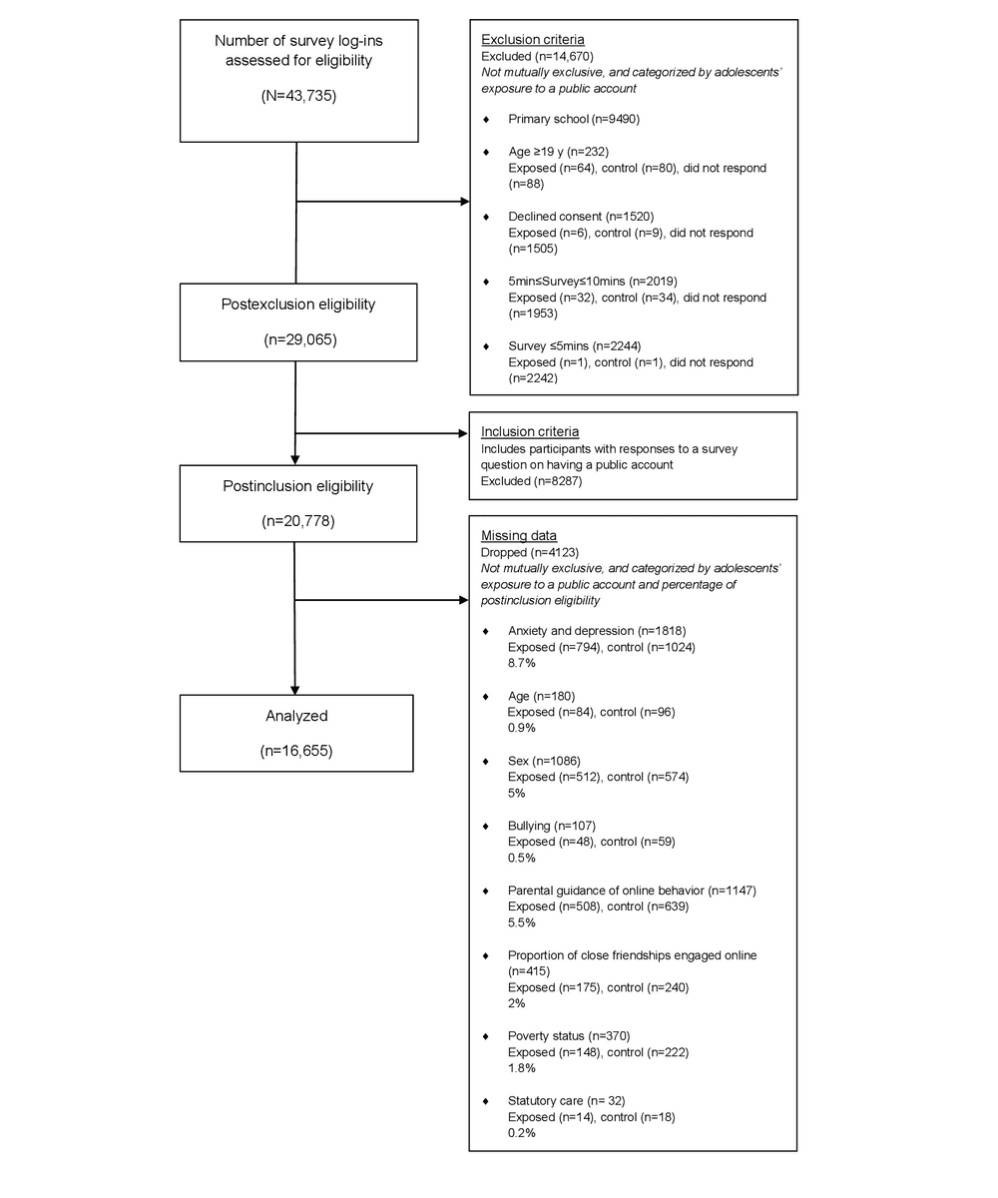
Flow diagram of participant eligibility.

The final study sample is defined as 16,655 adolescents in secondary schools and FECs aged 11 to 18 years responding to social media use questions. Adolescents were excluded from the sample if they spent <10 minutes on the survey as a minimum engagement threshold, ensuring validity in the integrity of the responses (Figure 1).

### Measures

#### Primary Exposure: Public Social Media Account

The primary exposure describes social media account privacy behavior in response to the question, “Do you have a publicly available social media account?” It is binarized into “having a publicly available social media account” defined as any account that does not require a follow request, friend request, or permission to access the account’s information, and “not having a publicly available social media account,” which includes “having a private social media account” requiring a follow request, friend request, or permission to access the account’s information [8].

#### Primary Outcome: Anxiety and Depression

The primary outcome in adolescents is “anxiety and depression,” a state of mental illness defined as being above the threshold for risk of clinical anxiety and depression using the Revised Child Anxiety and Depression Scale-11 (RCADS-11). The RCADS-11 includes 4-point scale items on depression (5 items) and anxiety (6 items), such as “I feel worthless” and “All of a sudden, I feel really scared for no reason at all.” A 2-item symptom impact score includes, “How much do these difficulties upset or distress you?” and improves screening sensitivity to 84% and specificity to 72%. The item scores align with the criteria of the *Diagnostic and Statistical Manual of Mental Disorders, Fifth Edition*. They are summed to produce a combined, internalizing symptom score for anxiety and depression that is binarized and interpreted at cutoffs of ≥14 for boys and ≥18 for girls [19].

#### Descriptive Variables: Social Media Use

Descriptive information on the distribution of SMPs and social media content accessed by adolescents was collected by asking, “In the last 24 hours, which online platforms and content have you accessed?” Responses to these questions provide a snapshot of the variability in adolescent social media use; platforms included YouTube, WhatsApp, Snapchat, TikTok, Instagram, Pinterest, BeReal, Discord, Twitter, Facebook, Reddit, Telegram, Omegle, and Signal, as well as “other,” and “none of these” options. Content response options included entertainment, humor, celebrities and influencers, family and friends, lifestyle, health and fitness, support and wellness, beauty and fashion, education, and forums, as well as “other” [8].

#### Covariates

##### Established Confounders

The International Alliance of Mental Health Research Funders recommends the use of RCADS-11 for the measurement of anxiety and depression in adolescents, with age and sex as demographic confounders [10,17,20]. In addition, the following a priori confounders are included: the experience of bullying [13], the perception of different parental guidance approaches to online behavior [14,15], the proportion of close friendships engaged with online [21-23], poverty status and exposure to placement in statutory care [13,24].

Bullying, especially online-mediated bullying (cyberbullying), is recognized as being particularly impactful on the mental health of adolescents, with girls being disproportionately affected [25]. Together with income inequality [25], as captured by poverty status in this study, these covariates contribute to significantly greater odds of negative moods and feelings in those who are bullied very often compared to those bullied less often [25] and poorer mental health symptoms overall [25,26]. Close friendships and the strength of these relationships are demonstrated to significantly predict adolescent mental health [21-23]. Greater proportional time spent engaging in these close friendships is demonstrated to impact young people’s mental health positively, with lower odds of negative moods and low self-esteem [13]. Parental guidance of online behavior captures adolescents’ perception of parental control or approaches to their online activities and their level of emotional closeness to their parents, which is shown to be a strong predictor of positive mental health [13]. Exposure to childhood maltreatment, expectedly, worsens mental health outcomes in young people and is represented in this analysis by adolescents who were exposed to being placed in statutory care [24].

Age and sex will be used in the analysis of a minimally adjusted model, and a priori confounders will be included in a fully adjusted model. Age, sex, and parental guidance of online behavior will be explored for effect modification of the relationship between social media privacy and anxiety and depression [14,15].

##### Confounder Categorization

The experience of bullying is categorized into 5 groups ranging from *never* to *every day*. Parental guidance of online behavior is categorized into 3 groups: *none*, *active guidance*, and *regulation*. *Active guidance* describes parents who “Encourage you to explore and learn things on the internet,” “Talk to you about what you do online,” “Explain why some websites are appropriate or inappropriate,” or “Help you when something bothers you on the internet.” *Regulation* describes parents who “Restrict your internet use,” “Log in to access your online social media accounts (eg, Facebook, Twitter, Instagram)” or “Monitor your internet use.” The proportion of close friendships primarily engaged with online is grouped into 3 categories in response to the question, “Out of all the individuals you would identify as close friends, how many do you primarily meet with online?”: *none*, *a few or some*, or *most or all.* Poverty status is binarized from responses to the question, “At home, I go to bed hungry because there is not enough food in the house.” Placement in statutory care is binarized from responses to the question, “Are you a child in care, a looked-after child, or fostered?”

### Statistical Analysis

#### Statistical Modeling

Data distributions are assessed for normality, and univariable association tests are performed between the primary exposure and outcome variables using crude logistic regression likelihood ratio tests (LRTs). Chi-square tests and ANOVA are used to independently test the primary exposure and outcome variables against a priori confounders and assess confounder collinearity. A multivariable logistic regression model is built to assess the association between having a public social media account and anxiety and depression, with stepwise inclusion of the confounders. LRTs are used to test categorical variables for departure from linearity, conduct sequential model adjustment for the strength of confounding, and assess for effect modification. Statistical significance is set at *P*<.05 using 95% CIs, and analyses are performed using Stata software (version 17.0; StataCorp LLC).

#### Sensitivity Analysis

A small proportion of adolescents (204/16,655, 1.22%) who report not using any survey-listed SMPs or any “other” SMPs not listed in the survey in response to a question that asks them to indicate which SMPs they had accessed in the past 24 hours are excluded from the full sample in a sensitivity analysis to determine whether the information they represent biases the observed associations between having a public social media account and anxiety and depression. This sensitivity analysis aims to achieve the closest comparison between adolescents with a public social media account and those without a public social media account who had a private social media account.

#### Power

Given that the estimated prevalence of probable mental disorders in UK adolescents ranges from 18% to 22% [27], to detect a 15% relative difference in the odds of depression and anxiety with a 2-tailed significance level of 95% and power of 80%, a total of 10,500 participants would be required for statistical analysis. Missing values independently constitute <10% of the selected covariates (Figure 1). Complete case analysis is performed to maintain statistical integrity accounted for by sufficient study power.

### Ethical Considerations

#### Ethics Approval

The study was approved by the University of Oxford Research Ethics Committee (R62366/RE0014). The research team provided all schools that signed up with detailed ethics procedures for the study, information for parents or guardians and pupils, and school-specific log-in IDs and passwords for their pupils.

#### Informed Consent

Participation in the OxWell survey was voluntary. Students aged <16 years were asked to assent to participation after their parents were given an opportunity to opt their child out of the study; those aged ≥16 years could consent to participation. Participants did not receive any incentives to take part in the study. Consent procedures are fully described in the protocol [18].

Pupils were informed and frequently reminded during the survey that they could stop at any time, leave questions blank, and move on to the next question if they did not wish to answer. At the end of the survey, pupils were advised what they could do if they felt worried or upset, including seeking support from available school staff. Information on appropriate online resources supporting adolescent mental health was also provided. If a survey response raised concern about a participant’s safety, the lack of identifiers in the survey meant that we could not positively identify pupils to provide direct referral to appropriate support.

#### Privacy and Confidentiality

Nonidentifiable data were collected without students’ names, addresses, or date of birth information. No unique individual log-ins were used, and IP addresses were not retained. The survey provider used encryption when transferring the data to the research team, and participants were instructed and reminded to close their browser window when they were finished.

## Results

### Participant Characteristics

The characteristics of 16,655 study participants are summarized and grouped by social media account (Table 1). Female individuals comprised 55.48% (9241/16,655) of all adolescents. The participants’ mean age was 13.8 (SD 1.84; range 11-18) years. Approximately two-thirds of the participants (10,860/16,655, 65.21%) fell into the young adolescent category (age 10-14 y), and the percentage of adolescents with a publicly available social media account was consistent across the age range with no statistically significant differences across the 2 age groupings (*P*=.84; Table 2). Of the 16,655 adolescents, 6734 (40.43%) reported having a public social media account. This group differed significantly from those without a public social media account across the remaining covariates, including age (*P*<.001; Table 3). Of the 16,655 adolescents, 5429 (32.6%) scored above the RCADS-11 threshold for risk of clinical anxiety and depression. A third of the adolescents (5511/16,655, 33.09%) reported experiencing *no parental guidance* over their online activities, while two-thirds (11,144/16,655, 66.91%) reported experiencing *active guidance* or *regulation*. One-fifth of the adolescents (3356/16,655, 20.15%) experienced all or most of their closest friendships online, while 35% (5836/16,655) reported experiencing none of their closest friendships online (Table 1).

**Table 1:** Participant characteristics by social media privacy.

Variables				Publicly available social media account				Total (n=16,655)		*P* value
				Yes (n=6734)		No (n=9921)				
**Continuous variables**										
	Age (y), median (IQR)			14.0 (12.0-15.0)		14.0 (12.0-15.0)		—^a^		—
	Age (y), mean (SD; range)			—		—		13.8 (1.84; 11.0-18.0)		—
**Categorical variables,** **n (%)**										
	**Anxiety and depression (RCADS-11)** ^b^									<.001
		Yes	2499 (37.11)		2930 (29.53)		5429 (32.6)			
		No	4235 (62.89)		6991 (70.47)		11 226 (67.4)			
	**Sex**									<.001
		Female	3293 (48.9)		5948 (59.95)		9241 (55.48)			
		Male	3441 (51.1)		3973 (40.05)		7414 (44.52)			
	**Bullying**									<.001
		Negligible	5291 (78.57)		8240 (83.06)		13,531 (81.24)			
		Occasionally	716 (10.63)		882 (8.89)		1598 (9.59)			
		Weekly	231 (3.43)		281 (2.83)		512 (3.07)			
		Frequently	318 (4.72)		366 (3.69)		684 (4.11)			
		Daily	178 (2.64)		152 (1.53)		330 (1.98)			
	**Parental guidance of online behavior** ^c^									<.001
		None	2508 (37.24)		3003 (30.27)		5511 (33.09)			
		Active guidance	2338 (34.72)		3241 (32.67)		5579 (33.5)			
		Regulation	1888 (28.04)		3677 (37.06)		5565 (33.41)			
	**Proportion of close friendships engaged with online** ^d^									<.001
		None	1892 (28.1)		3944 (39.75)		5836 (35.04)			
		A few or some	3244 (48.17)		4219 (42.53)		7463 (44.81)			
		Most or all	1598 (23.73)		1758 (17.72)		3356 (20.15)			
	**Poverty status**									<.001
		Yes	360 (5.35)		248 (2.5)		608 (3.65)			
		No	6374 (94.65)		9673 (97.5)		16,047 (96.35)			
	**Statutory care**									<.001
		Yes	470 (6.98)		462 (4.66)		932 (5.6)			
		No	6264 (93.02)		9459 (95.34)		15,723 (94.4)			

^a^Not applicable.

^b^RCADS-11: Revised Child Anxiety and Depression Scale-11 (symptom impact score: 0-39; positive anxiety and depression screen: ≥18 for girls, ≥14 for boys).

^c^Adolescent-reported parental approaches to their online activity.

^d^Adolescent-reported proportion of existing close friendships engaged with online.

**Table 2:** Percentage of adolescents with a publicly available social media account by age group and age.

		Publicly available social media account		Total (n=16,655), n (%)	*P* value	
		Yes (n=6734), n (%)^a^	No (n=9921), n (%)^a^			
**Age group (y)**						.84
	Young adolescents (10-14)	4397 (40.49)^b^	6463 (59.51)	10,860 (65.21)		
	Middle adolescents (15-19)	2337 (40.33)	3458 (59.67)	5795 (34.79)		
**Age (y)**						<.001
	11	507 (35.14)	936 (64.86)	1443 (8.66)		
	12	1257 (38.15)	2038 (61.85)	3295 (19.78)		
	13	1428 (42.7)	1916 (57.3)	3344 (20.08)		
	14	1205 (43.38)	1573 (56.62)	2778 (16.68)		
	15	995 (42.91)	1324 (57.09)	2319 (13.92)		
	16	748 (39.14)	1163 (60.86)	1911 (11.47)		
	17	414 (37.98)	676 (62.02)	1090 (6.54)		
	18	180 (37.89)	295 (62.11)	475 (2.85)		

^a^Row percentages derived from row totals.

^b^No age group differences in proportion of public accounts.

**Table 3:** Univariable associations between social media privacy and anxiety and depression and covariates.

			Chi-square (*df*)		*F* test (*df*)^a^		*P* value
**Publicly available social media account (binary)**							
	Anxiety and depression	104.2 (1)		—^b^		<.001	
	Age	52.3 (7)		—		<.001	
	Sex	198.4 (1)		—		<.001	
	Bullying	—		15.73 (4)		<.001	
	Parental guidance of online behavior	—		81.69 (2)		<.001	
	Proportion of close friendships engaged with online	—		130 (2)		<.001	
	Poverty status	92.4 (1)		—		<.001	
	Statutory care	41.0 (1)		—		<.001	
**Anxiety and depression (binary)**							
	Age	158.9 (7)		—		<.001	
	Sex	433.2 (1)		—		<.001	
	Bullying	1040.4 (4)		—		<.001	
	Parental guidance of online behavior	14.2 (2)		—		<.001	
	Proportion of close friendships engaged with online	26.5 (2)		—		<.001	
	Poverty status	339.3 (1)		—		<.001	
	Statutory care	21.3 (1)		—		<.001	

^a^ANOVA reporting.

^b^Not applicable.

### Social Media Use Patterns

The 5 leading SMPs accessed by adolescents in the previous 24 hours were YouTube, WhatsApp, Snapchat, TikTok, and Instagram (Table 4). Three-quarters of the adolescents (12,619/16,655, 75.77%) accessed entertainment content compared to 41.03% (6833/16,655) who engaged with content related to family and friends. Only 12.89% (2147/16,655) of the adolescents accessed support and wellness content, half of whom (1059/2147, 49.32%) screened positive for anxiety and depression (Table 5).

**Table 4:** Number of adolescents accessing social media platforms in the previous 24 hours proportioned by adolescents reporting having a publicly available social media account.

Social media platform	Total (N=16,655), n (%)	Publicly available social media account (yes; n=6734), n (%)	Proportion with public account (%)
YouTube	13,914 (83.53)	5762 (85.57)	41.41
WhatsApp	13,613 (81.68)	5554 (82.48)	40.8
Snapchat	11,595 (69.58)	5298 (78.66)	45.69
TikTok	11,552 (69.35)	5623 (83.5)	48.68
Instagram	9274 (55.68)	4358 (64.72)	46.99
Pinterest	4971 (29.85)	2099 (31.17)	42.22
BeReal	4839 (29.1)	2293 (34.05)	47.39
Discord	3889 (23.4)	2062 (30.62)	53.02
Twitter	3270 (19.63)	1911 (28.38)	58.44
Facebook	2454 (14.73)	1407 (20.89)	57.33
Other^a^	2065 (12.39)	1038 (15.41)	50.27
Reddit	1861 (11.17)	1026 (15.24)	55.13
Omegle	705 (4.23)	491 (7.29)	69.65
Telegram	375 (2.25)	242 (3.59)	64.53
Signal	215 (1.29)	117 (1.74)	54.42
None of these^b^	204 (1.22)	61 (0.91)	29.9

^a^Platforms accessed are not mutually exclusive: 2.08% (43/2065) of adolescents selecting “other” also selected “none of these.”

^b^None of these, n=204 are dropped in a sensitivity analysis.

**Table 5:** Number of adolescents accessing social media content in the previous 24 hours proportioned by adolescents screening positive for anxiety and depression.

Social media content	Total (N=16,655), n (%)	Anxiety and depression (yes; n=5429), n (%)	Proportion with anxiety and depression (%)
Entertainment	12,619 (75.77)	4218 (77.69)	33.43
Humor	10,467 (62.85)	3630 (66.86)	34.68
Celebrities and influencers	7323 (43.97)	2694 (49.62)	36.79
Family and friends	6833 (41.03)	2357 (43.41)	34.49
Lifestyle	5875 (35.27)	2223 (40.95)	37.84
Health and fitness	5669 (34.04)	2069 (38.11)	36.50
Beauty and fashion	5639 (33.86)	2341 (43.12)	41.51
Education	4643 (27.88)	1637 (30.15)	35.26
Other	4073 (24.46)	1259 (23.19)	30.91
Support and wellness	2147 (12.89)	1059 (19.51)	49.32
Forums	794 (4.77)	353 (6.50)	44.46

### Social Media Privacy: Associations

**Figure 2:**
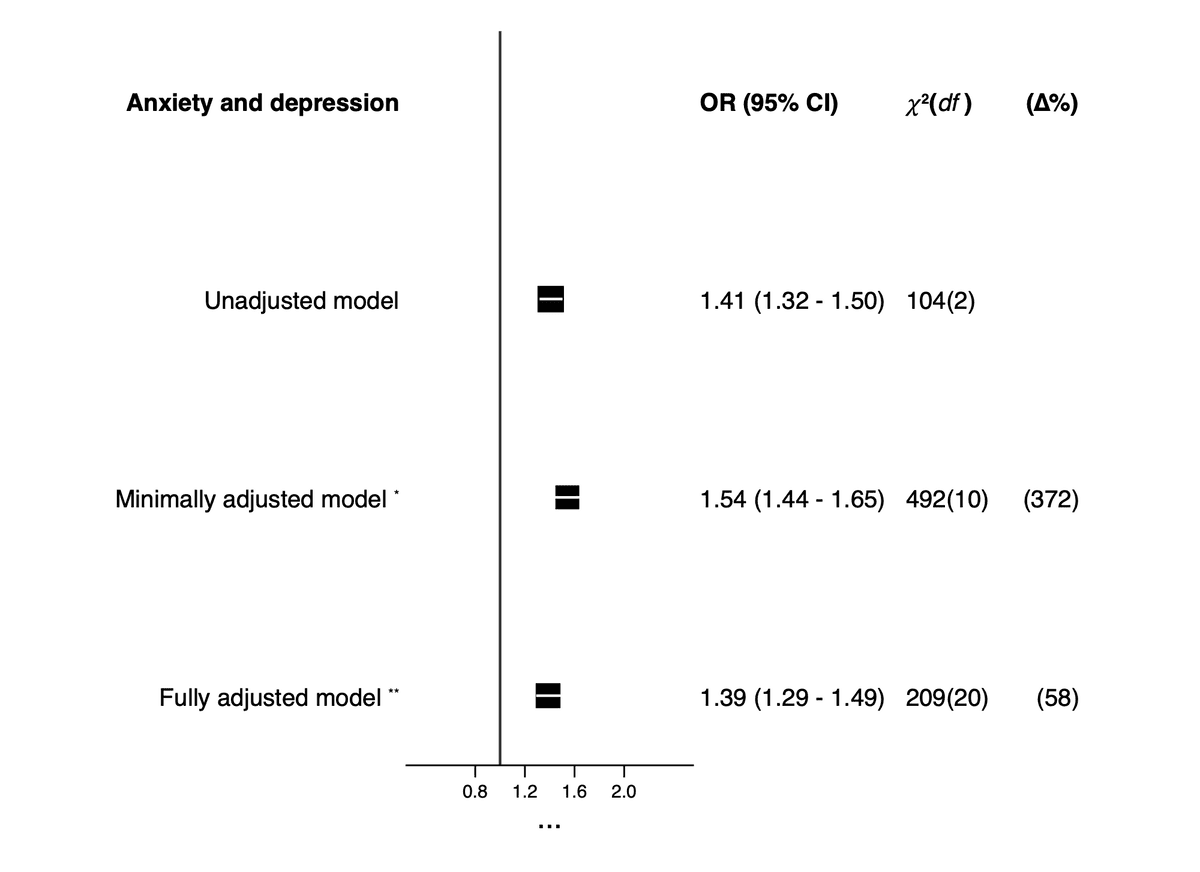
Forest plot showing the association between having a publicly available social media account and anxiety and depression using odds ratios (ORs) and 95% CIs comparing adolescents with a public social media account to those with no public social media account in 3 separately adjusted models. Likelihood ratio test for heterogeneity: *P*<.001. *Minimally adjusted model adjusted for age and sex. **Fully adjusted model adjusted for age, sex, bullying, parental guidance of online behavior, proportion of close friendships engaged with online, poverty status, and statutory care.

In an unadjusted model, adolescents with a publicly available social media account showed 41% higher odds of scoring above the threshold for the risk of clinical anxiety and depression compared to those with no public social media account (odds ratio [OR] 1.41, 95% CI 1.32-1.50; *P*<.001). In a model minimally adjusted for age and sex, this association was 13% higher (OR 1.54, 95% CI 1.44-1.65; *P*<.001). In a fully adjusted model, this association was attenuated to 39% higher odds of anxiety and depression in adolescents with a public social media account compared to those with no public social media account, controlled for age, sex, bullying, parental guidance of online behavior, proportion of close friendships engaged with online, poverty status, and statutory care (OR 1.39, 95% CI 1.29-1.49; *P*<.001; Figure 2). The LRT for heterogeneity showed that anxiety and depression outcomes differed significantly by social media account exposure (*P*<.001; Figure 2).

### Social Media Privacy: Effect Modification

**Figure 3:**
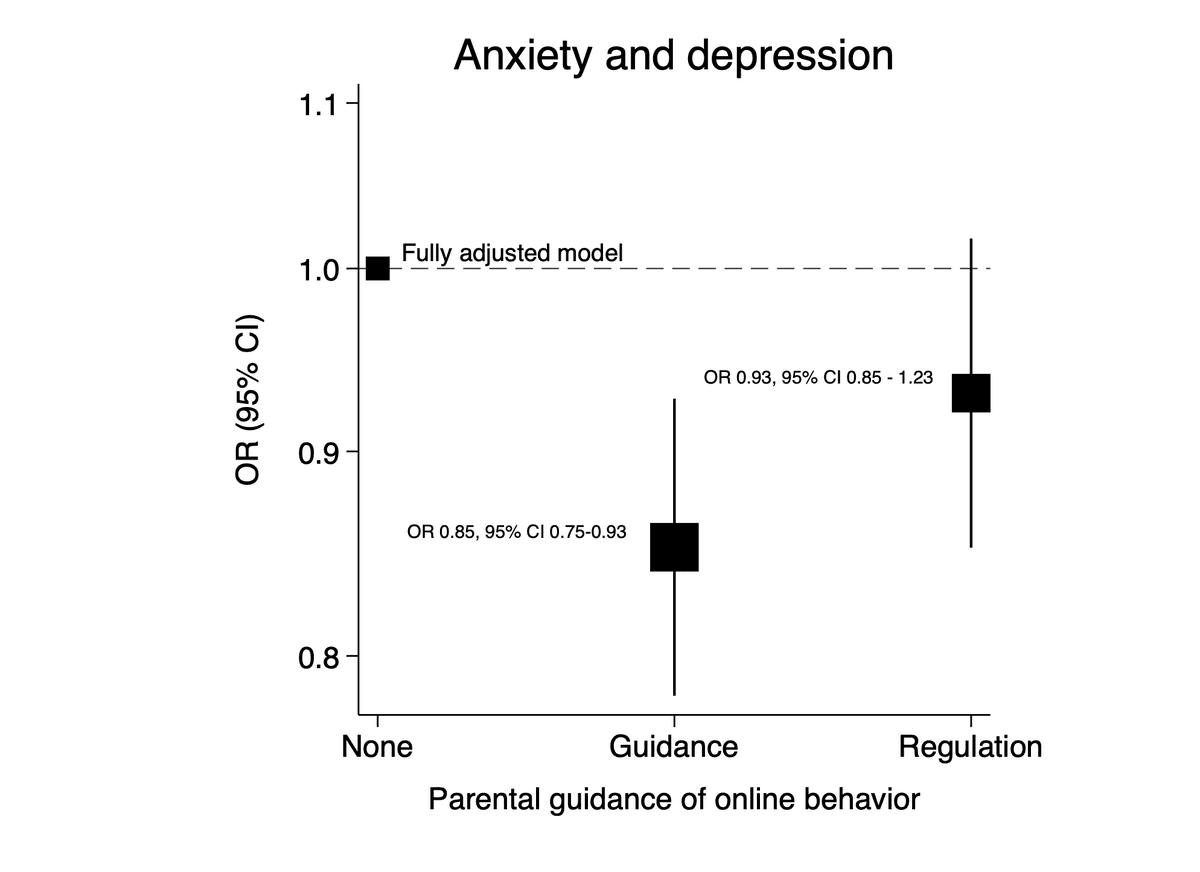
Shape plot showing the association between parental guidance of online behavior and anxiety and depression, accounting for social media account privacy, age, sex, bullying, proportion of close friendships engaged with online, poverty status, and statutory care. OR: odds ratio.

ORs for the association between anxiety and depression and social media privacy were not shown to be modified by age (*P*=.27) or sex (*P*=.34). However, ORs for the association between anxiety and depression and having a public social media account were found to be statistically significantly modified by parental guidance of online behavior (*P*=.007). Adolescents with a public social media account reporting either *active guidance* or *regulation* had 51% and 52% higher odds of anxiety and depression, respectively, compared to 18% higher odds of anxiety and depression in adolescents with a public social media account reporting *no parental guidance* (Table 6). Analysis in a fully adjusted model showed that, controlling for social media account exposure, adolescents reporting *active guidance* showed 15% lower odds of anxiety and depression compared to those reporting *no parental guidance* (*P*<.001). In adolescents reporting *regulation* compared to *no parental guidance*, the association was not statistically significant (*P*=.11; Figure 3).

**Table 6:** Odds ratios (ORs) for the anxiety and depression outcome in adolescents with a publicly available social media account compared to those without a public social media account, showing effect modification by parental guidance of online behavior.

Parental guidance of online behavior		OR (95% CI)	*P* value		LRT^a^ interaction chi-square (*df*)	
No publicly available social media account		Reference	—		—	
Publicly available social media account				.004		11.1 (2)
	No parental guidance	1.18 (1.04-1.33)	.007		—	
	Parental guidance	1.51 (1.34-1.71)	<.001		—	
	Parental regulation	1.52 (1.34-1.73)	<.001		—	

^a^LRT: likelihood ratio test.

### Sensitivity Analysis

By removing a small proportion of adolescents (204/16,655, 1.22%) who were most likely to represent those without a public social media account who had no social media account at all from the pool of those without a public social media account who had a private social media account, the analysis was able to demonstrate that there were no significant differences in the odds of anxiety and depression in a fully adjusted sensitivity analysis model (OR 1.38, 95% CI 1.28-1.48; *P*<.001; Multimedia Appendix 1) compared to a fully adjusted model in the full sample (Figure 2).

**Figure 4:**
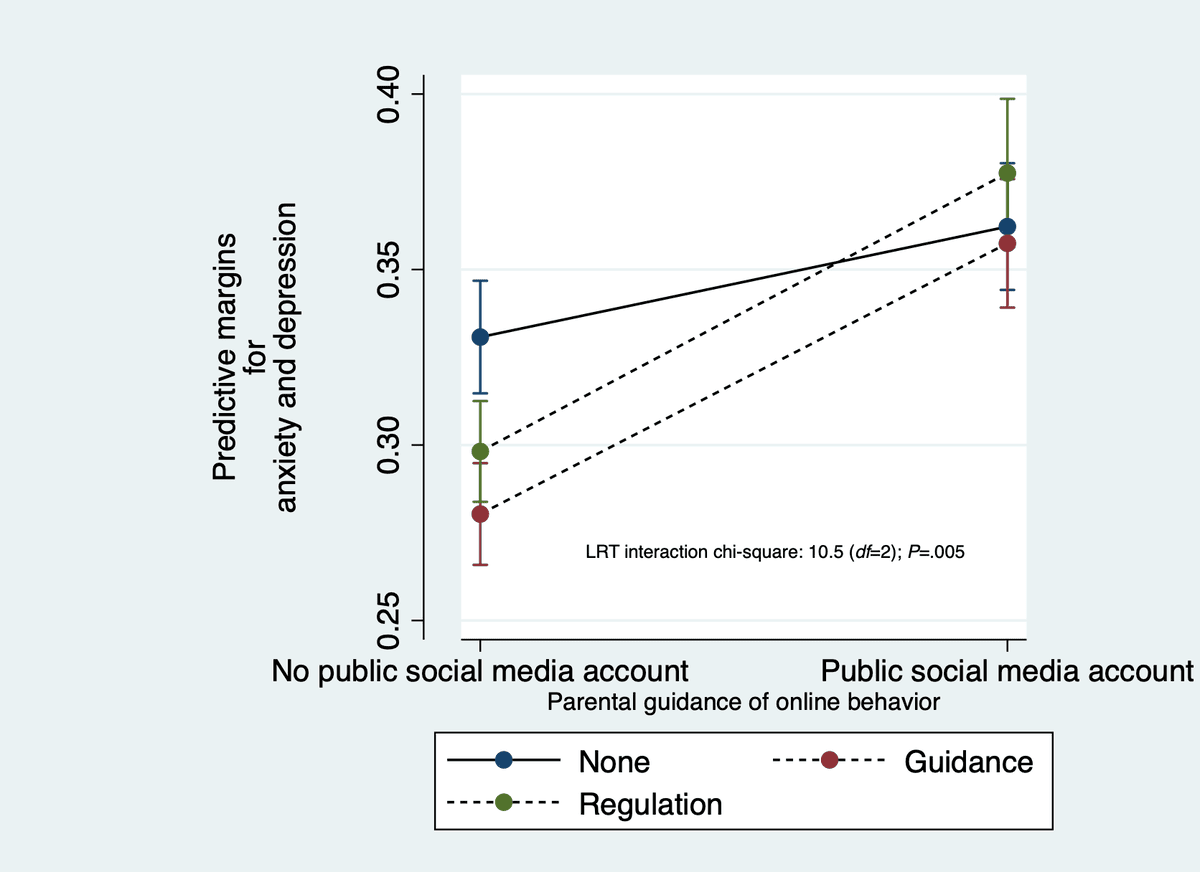
Predictive margins plot from the sensitivity sample showing effect modification of social media account privacy by parental guidance of online behavior on the probability of anxiety and depression. LRT: likelihood ratio test.

**Figure 5:**
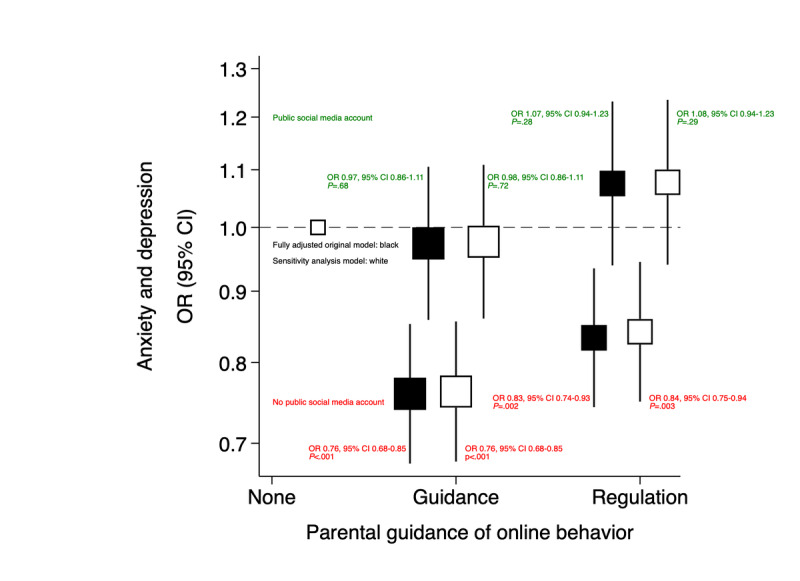
Shape plot of original sample and sensitivity analysis sample showing effect modification of the association between parental guidance of online behavior and anxiety and depression by social media account. OR: odds ratio.

The effect modification of parental guidance of online behavior on the association between having a public social media account and anxiety and depression also remained significant (*P*=.005) in a fully adjusted sensitivity analysis model (Multimedia Appendix 2), and predictive margins demonstrated that the greatest differences in anxiety and depression outcomes were observed between adolescents who were not exposed to having a public social media account and who reported no parental guidance of their online behavior compared to those who received *active guidance* or *regulation* (Figure 4). This was supported by comparable effect modification associations between the full sample and the sensitivity analysis sample (Figure 5).

## Discussion

### Principal Findings

This study set out to describe the association of social media account privacy, determined by whether participants had a public social media account or no public social media account, and symptoms of clinical anxiety and depression in a population of 16,655 school-going adolescents. We found that adolescents with a public social media account had 1.39 times higher odds of symptoms of anxiety and depression compared to those with no public social media account. This association suggests that having a public social media account is likely to be an important factor in the complex behavioral exposure of social media use and should be incorporated in guidance pertaining to online behaviors. We also found that parental approaches to their adolescents’ online activity are likely to be an important factor in their mental health.

One interpretation is that adolescents without a public social media account maintain some control over the amount and quality of the material they share publicly, as suggested by Gruzd and Hernández-García [5], and possibly exercise greater awareness of the nature of the material that they, in turn, are exposed to online. A potentially beneficial role of such self-regulation is echoed in findings from an experimental study demonstrating how a 4-week randomized, financially compensated Facebook deactivation intervention increased offline activities among participants [28]. This resulted in increased personal social activity with family and friends, as well as improved depression and anxiety, with an effect equivalent to a quarter of that achieved by psychological therapy at the most conservative estimate [28,29].

In a study investigating specific online disclosure behaviors, nearly half of the social media users reported difficulty managing their privacy settings [5]. The more an individual was concerned about “horizontal” privacy threats from peer group social contacts, the greater the amount and depth of their online disclosures and the weaker the accuracy and awareness of their online disclosures, specifically describing the intentional awareness of self-disclosing online. The more an individual was concerned about “vertical” privacy threats from hierarchical corporations, the greater the accuracy and awareness of their online disclosures and the weaker the amount and depth of their online disclosures; private social media account holders in this group were associated with having more positive content in their online disclosures [5], partially supported by evidence of lower odds of anxiety and depression in adolescents who did not have a public social media account from our findings.

### Social Media Privacy Interaction With Parental Guidance of Online Behavior

The association between social media privacy and anxiety and depression was, of interest, only modified by parental guidance of online behavior, characterizing parents who discuss concepts around the risks of online activity as providing *active gui*dance and parents who monitor their children’s online activity as providing *regulation* [14,15]. Adolescents with either *active guidance* or *regulation* had approximately 50% higher odds of anxiety and depression compared to 18% higher odds in adolescents with a public social media account reporting *no parental guidance*. One interpretation of this finding suggests that adolescents experiencing anxiety and depression may have reported behavior from their caregivers that indicated caregiver recognition of their adolescent’s mental distress, prompting the implementation of parental guidance and regulation approaches in addressing what may have been perceived by caregivers as the exacerbating effect of their children’s social media use on anxiety and depression. This interpretation is supported by analysis showing 15% lower odds of anxiety and depression in adolescents reporting *active guidance* compared to those adolescents reporting *none* (*P*<.001), with no significant effect in those reporting *regulation* (*P*=.45).

Interestingly, upon closer examination of the interaction between social media privacy and parental guidance of online behavior, it was shown that regulatory parental guidance also decreased the odds of anxiety and depression compared to no parental guidance, but only in adolescents who did not have a public social media account (*P*=.002), and not in adolescents with a public social media account *(P*=.28; Figure 5)*.* This reduction was also statistically significant (*P=*.003) in a sensitivity analysis after excluding adolescents who reported not accessing any SMP in the previous 24 hours (Figure 5). Strict regulation may demonstrate lesser benefit in adolescents with a public social media account despite probably intending to reduce the amount of social media use, while open discussion might cautiously be interpreted to be more effective in influencing adaptive online behaviors.

An equally plausible interpretation is that parents who are more regulatory in their approach may be doing so in response to symptomatic mental illness, such as observing severe depressive episodes or self-harming behaviors in their children or responding to concern over their children’s disclosure of information online. The relationship between parental guidance of online behavior and anxiety and depression can only be clarified with longitudinal data and in their present form generate the basis of valuable hypotheses. One such valuable association hypothesis is that parents demonstrating *active guidance* may enjoy, on average, healthier relationships with their children, potentially conferring a decreased odds of anxiety and depression risk due to the quality of their interpersonal relationship captured within the variable [6].

### Social Media Use Patterns

The primary descriptive analysis of social media use patterns found that YouTube, WhatsApp, and Snapchat were the most accessed SMPs in the previous 24 hours. Adolescents’ preferred SMP activities settled on video and direct messaging formats, while global social media use statistics in 2023 ranked Facebook, YouTube, and WhatsApp as the top SMPs [30]. This highlights shifting generational priorities, with only 14.7% (2454/16,655) accessing Facebook in our study [30]. Almost 49.32% (1059/2147) of the adolescents accessing support and wellness content (2147/16,655, 12.89% of the sample accessed this content) were at risk of anxiety and depression. Given curiosity about the role that online interventions might be able to play, our findings indicate that service uptake in support and wellness is among the least accessed categories, markedly lower than the 32.6% (5429/16,655) of adolescents at risk for anxiety and depression—consistent with previous OxWell data findings about limited online support sought after self-harm [31].

Of the 16,655 adolescents, 9921 (59.57%) reported having no public social media account. New developments in the social media space may complicate the interpretation of such observations. The Threads platform, released in July 2023 by Meta, delivers an unfiltered feed of information that users cannot control while allowing them not to make their accounts public. We cannot know if associations between having a public social media account and anxiety and depression are driven by what young people share versus what they see on their feeds—and future studies should try to disentangle this experimentally. The blurring of public and private social media account features is a rising trend among SMPs. It demonstrates how notions of private and public are becoming less binary, intended by SMPs pursuing behavioral reinforcement through app engagement that adolescents are more vulnerable to. This foregrounds the case for generating useful evidence in sustaining the momentum behind policy-backed social media accountability and mitigation frameworks [5].

### Ethical Accountability

A helpful approach in conceptualizing frameworks of responsibility for SMPs and the potential implications of these technologies on adolescents as a vulnerable group draws elements from ethical scholarship. Artificial intelligence algorithms have captured the public imagination. Their processing power exceeds human capabilities, leaving ample room for the application of ethical oversight. To satisfy demands for accountability, SMPs should operate with the understanding that they know what they are doing as agents of responsibility. This condition is presupposed by the moral requirement to provide reasons for their actions to those to whom they are answerable, which, crucially, includes adolescent users who are demonstrably vulnerable to the effects of their algorithms. Recent artificial intelligence algorithmic administration seems to mystify the human agents behind their implementation, thus necessitating a transfer of responsibility from the technology itself to the humans in charge. Coeckelbergh [32] describes how giving the right of explanation rather than the right to information can be more beneficial because it places the onus on SMPs to package such explanations in response to adolescent concerns against growing policy pressures.

Stakeholder involvement has been integral to collecting data within the OxWell study as a model for building trust with adolescents, their caregivers, and school communities. This approach creates the necessary scaffolding for the potential cocreation of online privacy literacy among adolescents [33]. In 2023, the American Psychiatric Association published a health advisory on social media use in adolescence, recommending that in young adolescents, adult monitoring by way of ongoing review, discussion, and coaching around social media content is advised for most youths’ social media use and that their autonomy may increase as they age and gain digital literacy [34]. However, this should be balanced with youths’ appropriate needs for privacy. Recent policy developments have culminated in parallel bills introduced in the US Senate and the UK Parliament. The US Protecting Kids on Social Media Act would restrict children aged <13 years from creating social media accounts and require parental consent for teenagers aged <18 years to sign up for SMPs [35]. The UK Online Safety Act, which was passed in 2023, recognizes age groups judged to be at risk of harm by SMP features, requiring SMPs to institute platform functionalities allowing for control over the content encountered by children [36].

### Limitations

This study has certain limitations. Findings from cross-sectional studies cannot determine the causal inference of association, which remains beyond the scope of this study design. The multilevel structure of data collected from different schools in this study may have benefited from multilevel analytical techniques suited to instances of data of this nature—beyond this study’s appropriately used pooled, regression-based analytical techniques. In addition, a distinct exposure classification might have included a third category accounting for the small proportion of adolescents (204/16,655, 1.22%) who reported not accessing SMPs in the past 24 hours, with statistical analysis using multiordinal regression techniques; however, because of predicted challenges with the use of underpowered categorical variables, we excluded these participants from analysis in a sensitivity analysis that showed no significant differences against all hypothesized associations. Observed association risks between individuals with a public social media account versus those without a public social media account and anxiety and depression do not necessarily represent associative risk within the same individual over time, demonstrating the need for a cautious interpretation of the behavioral social media account phenotype’s effect as being confined to the point in time at which it was recorded.

Spurious findings from subgroup analyses are not infrequent among well-powered studies, and the magnitude of ORs in our analysis reflects our primary outcome’s high prevalence at 32.6% (5429/16,655) screening positive for anxiety and depression. These ORs tend to be higher than the absolute effect size. Not all plausible confounders are accounted for in the regression model due to limitations arising from the characterization of legacy variables in the original dataset of the OxWell Student Survey. However, our conservative approach toward including a priori confounders was balanced against the risk of residual confounding. Plausible confounders for inclusion in future work include parental educational attainment as a gold standard measure for socioeconomic status and school deprivation indices as a measure of the availability of school resources in addition to parental factors.

The OxWell Student Survey could not determine an overall response rate due to the study team’s student recruitment pathway through local education authorities and schools according to a prespecified data protection protocol. However, diverse representation in the study is considerably strengthened by the breadth of exposure to 80 geographically and economically varying schools from the south and north of England, spanning 8 local education authorities. Only a proportion of the eligible sample (16,655/29,065, 57.3%) was included in this study. A fair proportion of the students (8287/29,065, 28.51%) had to be excluded because they did not answer the question on their type of social media account, which appeared further along the survey for which students might not have been allocated sufficient time. The study sample had 90% power with the sample size of 16,655 students, drawn from a considerably representative pool, demonstrating plausible generalizability to an English population of *young* and *middle adolescents*.

### Conclusions

The findings from this study confirm the dynamic nature of social media use, specifically highlighting that adolescents with a public social media account may face a higher behavioral risk for anxiety and depression than those without a public social media account. These findings highlight the future role of parental guidance of online activity in supporting the mental health of all adolescents, especially those with a public social media account, and demonstrate how adolescent perceptions of how their parents approach their online activity is associated with significant reductions in the odds of anxiety and depression among young people who do not have a public social media account. Official recommendations in keeping with these findings have been published by the American Psychiatric Association, and attempts to regulate SMPs targeting adolescents are actively underway [34-36]. Social media behaviors and associated patterns of use are complex, and determining which specific behaviors are likely to be associated with risk can help us better understand how best to inquire about and understand the impact of social media use on adolescent mental health.

### Future Work

Our findings, along with those of prior researchers, are exploratory steps highlighting the complexity of the behavioral mechanisms at play within social media use. Future research using experimental and longitudinal designs to investigate causal differences between additional behavioral phenotypes, including adolescents with both public and private social media accounts and those without social media accounts, and the resulting impact on mental illness may benefit from the application of network and factor analytic methodology in addressing the complexity of this primary exposure. The value of elucidating parental approaches to the guidance of adolescents’ behaviors on SMPs also emerges as a parallel feature that may prove increasingly important. Details on the motivations that parents and caregivers report behind their specific guidance of adolescent online behaviors become key. None of this should be attempted without the coproduction of research questions and the investigation of social media phenomena that are relevant to adolescents, both as a source of insight into adolescent needs and behaviors and as a source of clarity in interpreting findings emerging from such research.

In conclusion, including social media account privacy behaviors and further phenotypes in social media and digital mental health analysis associations promises to contribute to an increasingly detailed picture of this multidimensional, exposure-outcome relationship.

## Appendix

Multimedia Appendix 1 Sensitivity analysis showing odds ratios for anxiety and depression outcomes in adolescents who have a publicly available social media account compared to adolescents without a public social media account.

Multimedia Appendix 2 Sensitivity analysis showing effect modification of having a public social media account by online parental guidance on anxiety and depression outcomes.

## Abbreviations

FEC: further education college
LRT: likelihood ratio test
OR: odds ratio
RCADS-11: Revised Child Anxiety and Depression Scale-11
SMP: social media platform

## Data Availability

The datasets generated during and analyzed during this study are not publicly available due to ethical and information governance restrictions. To ensure that uses of the data fall under the remit of the intended purposes set out in the privacy information and to prevent duplication of analyses, data are available from the research team on reasonable request after a review process. The full list of questions as well as other details are available on a project-specific OxWell Open Science Framework website [37] along with the preregistration of the study protocol [38].
