# Supplementary material for: Exploring the Relationship Between Public Social Media Accounts, Adolescent Mental Health, and Parental Guidance in England: Large Cross-Sectional School Survey Study
Source: J Med Internet Res. 2024 Dec 17;26:e57154. doi: 10.2196/57154 (PMC11688589; doi:10.2196/57154)
Supplement: Multimedia Appendix 1 [file jmir_v26i1e57154_app1.docx]

Multimedia Appendix 1. Sensitivity analysis showing odds ratios for anxiety and depression outcomes in adolescents who have a publicly available social media account compared to adolescents who have no public account.

| **Hierarchical logistic regression model** | OR | SE | *P value* | 95% CI | LRT^a^  model fit chi-square (*df*) | Pseudo R^2^ | LRT ^b^ heterogeneity chi-square (*df*) |
| --- | --- | --- | --- | --- | --- | --- | --- |
|  |  |  |  |  |  |  |  |
| **Baseline model association:**  **Public account x Anxiety and depression** | 1.39 | .047 | < .001 | (1.30, 1.49 ) | 2021 (7) | .0047 | 97.1  (1) |
| **Minimally adjusted model:**  **Public account x Anxiety and depression**  *Age + Sex*  *LRT Heterogeneity* 𝜒2=*150.4*  *p<.001* | 1.53 | .053 | < .001 | (1.43, 1.64 ) | 1379.5 (5) | .0355 | 738.6 (3) |
| **Fully adjusted model:**  **Public account x Anxiety and depression**  *Age + Sex,*  *+ Bullying,*  *+ Parental guidance of online behavior,*  *+ Proportion of close friendships engaged online,*  *+ Poverty status,*  *+ Statutory care*  *LRT Heterogeneity* 𝜒2=*75.1*  *p<.001* | 1.38 | .051 | < .001 | (1.28, 1.48 ) | —^c^ | .1018 | 2118 (13) |

**^a^**LRT: likelihood ratio test for model fit. The reduced models significantly, better explain the variance in the outcome of anxiety and depression compared to the fully adjusted model.

**^b^**LRT: likelihood ratio test for heterogeneity. Anxiety and depression outcomes significantly differ by the exposure of having a public social media account.

**^c^**Not applicable.
