# Supplementary material for: Exploring the Relationship Between Public Social Media Accounts, Adolescent Mental Health, and Parental Guidance in England: Large Cross-Sectional School Survey Study
Source: J Med Internet Res. 2024 Dec 17;26:e57154. doi: 10.2196/57154 (PMC11688589; doi:10.2196/57154)
Supplement: Multimedia Appendix 2 [file jmir_v26i1e57154_app2.docx]

Multimedia Appendix 2. Sensitivity analysis showing effect modification of having a public account by parental guidance of online behavior, on anxiety and depression outcomes.

| **Anxiety & Depression** | | **Odds Ratio** | ***P* value** | **LRT^a^ interaction chi-square (*df*)** |
| --- | --- | --- | --- | --- |
|  |  |  |  |  |
| **Publicly Available Social Media Account** |  |  |  |  |
| *No* |  | 1 |  |  |
| *Yes* |  |  |  |  |
|  | ***Parental Guidance of Online Behavior*** |  | .005 | 10.6 (2) |
|  |  |  |  |  |
|  | None | 1.17 (1.04 - 1.32) | .01 | —^b^ |
|  | Parental Guidance | 1.50 (1.33 - 1.70) | < .001 | — |
|  | Parental Regulation | 1.50 (1.32 - 1.71) | < .001 | — |
|  |  |  |  |  |

^a^LRT: likelihood ratio test.

^b^Not applicable.
